# Adult expression of a 3q13.31 microdeletion

**DOI:** 10.1186/1755-8166-7-23

**Published:** 2014-03-20

**Authors:** Chelsea Lowther, Gregory Costain, Rebecca Melvin, Dimitri J Stavropoulos, Anath C Lionel, Christian R Marshall, Stephen W Scherer, Anne S Bassett

**Affiliations:** 1 Clinical Genetics Research Program, Centre for Addiction and Mental Health, Toronto, ON, Canada; 2 Institute of Medical Science, University of Toronto, Toronto, ON, Canada; 3 Cytogenetics Laboratory, Department of Pediatric Laboratory Medicine, Hospital for Sick Children, Toronto, ON, Canada; 4 Department of Laboratory Medicine and Pathology, University of Toronto, Toronto, ON, Canada; 5 The Centre for Applied Genomics and Program in Genomics and Genome Biology, The Hospital for Sick Children, Toronto, ON, Canada; 6 Department of Molecular Genetics and McLaughlin Centre, University of Toronto, Toronto, ON, Canada; 7 Department of Psychiatry, University of Toronto, Toronto, ON, Canada

**Keywords:** 3q13 deletion, Schizophrenia, Copy number variation, Genotype-phenotype correlation, Genetic counselling, Genomic disorder, Nonverbal learning disability

## Abstract

**Background:**

The emerging 3q13.31 microdeletion syndrome appears to encompass diverse neurodevelopmental conditions. However, the 3q13.31 deletion is rare and few adult cases have yet been reported. We examined a cohort with schizophrenia (n = 459) and adult control subjects (n = 26,826) using high-resolution microarray technology for deletions and duplications at the 3q13.31 locus.

**Results:**

We report on the extended adult phenotype associated with a 3q13.31 microdeletion in a 41-year-old male proband with schizophrenia and a nonverbal learning disability. He was noted to have a speech impairment, delayed motor skills, and other features consistent with the 3q13.31 microdeletion syndrome. The 2.06 Mb deletion overlapped two microRNAs and seven RefSeq genes, including *GAP43*, *LSAMP, DRD3*, and *ZBTB20.* No overlapping 3q13.31 deletions or duplications were identified in control subjects.

**Conclusions:**

Later-onset conditions like schizophrenia are increasingly associated with rare copy number variations and associated genomic disorders like the 3q13.31 microdeletion syndrome. Detailed phenotype information across the lifespan facilitates genotype-phenotype correlations, accurate genetic counselling, and anticipatory care.

## Background

Preliminary evidence suggests that individuals with microdeletions at chromosome 3q13.31 (OMIM #615433) may be predisposed to a broad spectrum of neurodevelopmental/neuropsychiatric conditions including global developmental delay/intellectual disability (DD/ID), speech delay, autism spectrum disorder (ASD), and attention deficit hyperactivity disorder (ADHD) [1-5]. Inconsistent molecular and cytogenetic techniques as well as variable genomic breakpoints have made pinpointing the responsible gene(s) within the 3q13.31 region challenging. In a large case series, Molin et al. defined a 580 kb shortest region of overlap (SRO) that includes five RefSeq genes: *DRD3*, *ZNF80*, *TIGIT*, *MIR568*, and *ZBTB20*[1]. Those authors proposed that haploinsufficiency of *DRD3* (OMIM #126451), which codes for the D3 subunit of the dopamine receptor, and *ZBTB20* (OMIM #606025), a DNA transcription repressor expressed in hippocampal neurons, may contribute to the neurodevelopmental features of the 3q13.31 microdeletion syndrome [1,6]. Recently, a 1.3 Mb deletion at 3q13.31 downstream of this proposed SRO and encompassing just two genes (*LSAMP*, *GAP43*) was identified in a 7-year-old female with ADHD, hypotonia, and postnatal growth above the mean [3]. *LSAMP* (OMIM #603241) encodes a limbic system-associated membrane protein (LAMP) and has been shown to regulate anxiety-like phenotypes in mice [7]. *GAP43* (OMIM #162060) is almost exclusively expressed in neuronal tissue and is a candidate gene for ASD [8].

Later-onset conditions, including neurodevelopmental diseases like schizophrenia, may also be associated with rare copy number variations (CNVs) [9]. To date, few adult cases with 3q13.31 deletions have been described in the literature, highlighting the paucity of phenotypic data across the lifespan needed to inform genetic counselling and anticipatory care. Here we provide a description of the extended adult phenotype associated with a 3q13.31 microdeletion [9] that encompasses all four of the previously proposed neurodevelopmental candidate genes at this locus: *DRD3*, *ZBTB20*, *LSAMP*, and *GAP43*.

## Case presentation

### Clinical description

**Figure 1:**
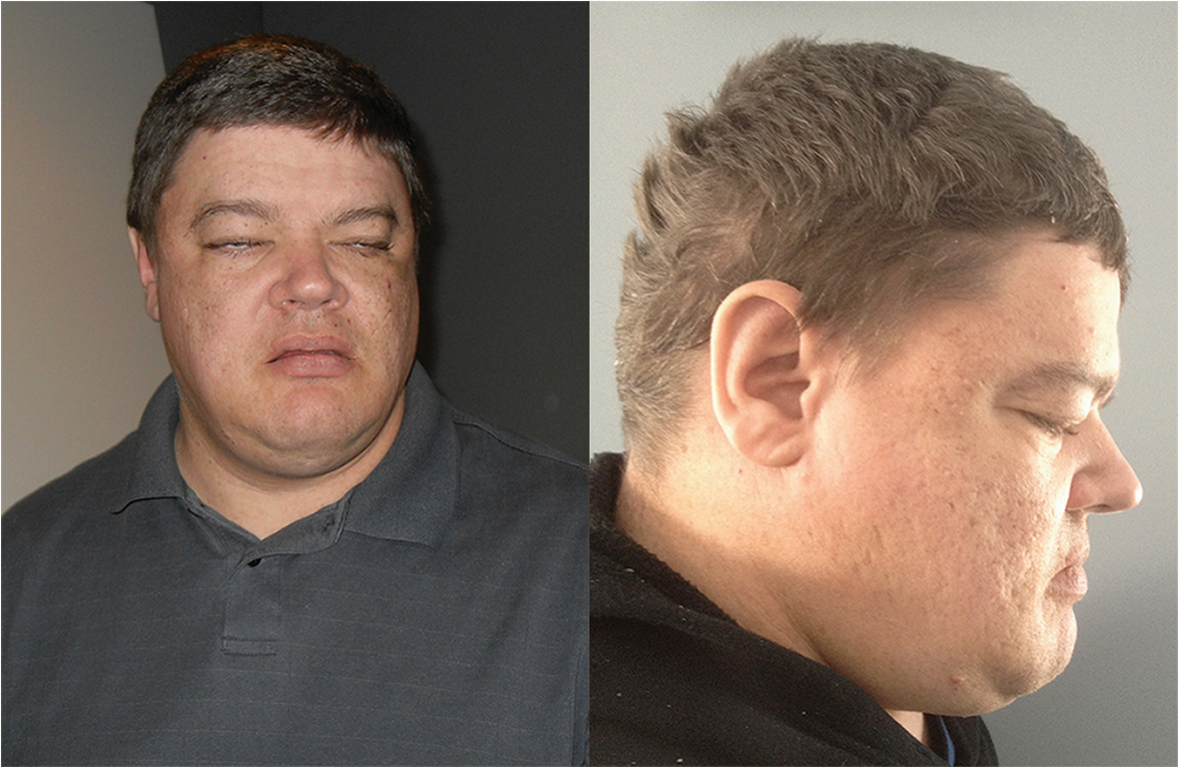
**Physical features of an adult male with a 3q13.31 microdeletion.** Anthropometric data demonstrated a post-natal growth pattern above the mean: at 31 years height was 181.5 cm (75^th^-90^th^ percentile), weight was 90.9 kg (BMI 27.6), and OFC was 60.9 cm (97^th^ percentile). Findings noted on physical examination as an adult included a broad neck and slight kyphosis. Craniofacial features included facial asymmetry, strabismus, narrow palpebral fissures, a long philtrum, a narrow high arched palate, flat occiput, and frontal and occipital hair whorls. He also had bilateral cubitus valgus, short 4^th^ and 5^th^ metacarpals bilaterally, as well as narrow feet with mild clinodactyly bilaterally of all toes and camptodactyly bilaterally of 4^th^ and 5^th^ toes.

The male proband (Figure 1) was conceived naturally to non-consanguineous parents of European ancestry, a 29-year-old mother and 36-year-old father. There was no known family history of major developmental or neuropsychiatric conditions. The pregnancy and term delivery were unremarkable; birth weight was 3,941 g (75^th^-90^th^ percentile). There was no evidence of hypotonia. The patient was noted to have delayed motor developmental milestones and “clumsiness” prior to age 3 years, when he was hospitalized for suspected viral meningoencephalitis with decreased level of consciousness and dystonic movements. A speech impairment and enuresis was noted thereafter and persisted into adulthood. At age 9 years, fine motor and perceptual motor skills were noted to be under-developed. A head CT (computed tomography) scan at age 15 years showed focal hypoplasia of the superior cerebellar vermis; a repeat CT scan at age 33 years was read as normal.

He was enrolled in a special education program beginning at age 7 years, with particular difficulties noted in mathematics and writing. At age 33 years, clinical neuropsychological testing revealed a full-scale IQ in the borderline range. Research-based testing at age 39 years using the WASI (Wechsler Abbreviated Scale of Intelligence) demonstrated a full-scale IQ of 75, with a marked difference between verbal (90) and performance (62) scores consistent with a nonverbal learning disability.

The patient developed schizophrenia and was first treated with standard antipsychotic medications at age 24 years. Overall, he has had an unremarkable course of illness (details available upon request); of note, aggressive behaviour at age 28 years prompted a change in his medication regimen. His past medical history also includes type 2 diabetes mellitus treated with insulin and hypercholesterolemia diagnosed in his 20′s, mild hypocalcemia first identified at age 31 years, bilateral L5-S1 disc protrusion requiring surgery at 34 years, and hypertension diagnosed at age 34 years. At time of last contact, he was 41 years old and living in a supported situation in the community.

### Molecular studies

**Figure 2:**
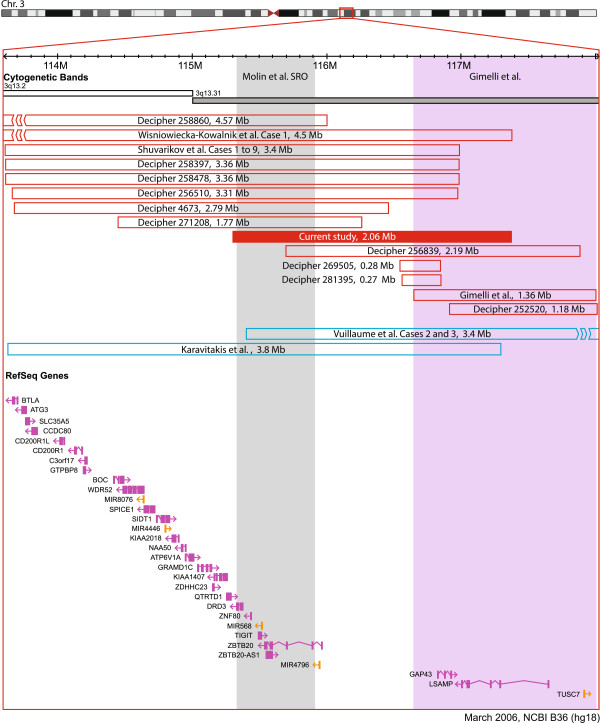
**3q13.31 deletions and duplications overlapping neuropsychiatric candidate genes*DRD3*,*ZBTB20*,*LSAMP,*and*GAP43.*** The image was modified from the Database of Genomic Variants (http://dgv.tcag.ca), NCBI Build 36 (hg 18) [13]. Only deletions and duplications <5 Mb in size and overlapping at least one of *DRD3*, *GAP43*, *ZBTB20*, and *LSAMP* are shown. Deletions and duplications are represented by red and blue bars, respectively. All genes (purple) and miRNAs (orange) within the 3q13.2-q13.31 region are shown; splice variants are not included. The grey bar outlines the coordinates for the 580 kb (chr3:115,335,356-115,916,848) shortest region of overlap (SRO) defined by Molin et al. [1]. The purple bar outlines the boundaries for the Gimelli deletion (chr3:116,640,577- 118,002,810, hg 18) that overlapped *GAP43* and *LSAMP*[3]. The 4.5 Mb deletion reported by Wisniowiecka-Kowalnik [14], the 4.57 Mb deletion in DECIPHER Case 258860, and the 3.4 Mb duplication by Vuillaume [4] extend beyond the boundary of the figure and are represented by line breaks. Where necessary, all deletion and duplication coordinates (i.e., excluding the Molin et al. SRO and the case described in this report) were converted from hg 19 to hg 18 using The UCSC Genome LiftOver tool (http://genome.ucsc.edu).

The patient was recruited into a longitudinal study of the genetics of schizophrenia [9] at age 31 years. Research chromosomal microarray analysis using the Affymetrix® Genome-Wide Human SNP Array 6.0 ultimately demonstrated a deletion at cytoband 3q13.31 (chr3:115,308,450–117,370,859, hg18) [9], which was clinically confirmed at a CLIA (Clinical Laboratory Improvement Amendments) approved laboratory and reported back to the patient. All stringent genome-wide CNV calls in this individual are included in Additional file 1: Table S1 with only the 3q13.31 deletion predicted to be pathogenic [10]. As is common with adult-onset disorders like schizophrenia, both parents were unavailable for testing (deceased). The deletion overlaps two microRNAs (miRNAs) and seven RefSeq genes (Figure 2), including the four promising neurodevelopmental gene candidates highlighted by Gimelli et al. (*GAP43* and *LSAMP*) and Molin et al. (*DRD3* and *ZBTB20*) [1,3]. The 3q13.31 region was inspected for CNVs in the DECIPHER (http://decipher.sanger.ac.uk) database and 10 patients were found to have deletions overlapping the one presented in this case report (Figure 2) [11]. In the 26,826 adult control subjects with genome-wide CNV data available to our colleagues at The Centre for Applied Genomics (TCAG), no similar 3q13.31 deletions or duplications were identified using a 50% reciprocal overlap criterion (Additional file 2: Table S2) [9,12].

## Discussion

### 3q13.31 deletion expression in adults

Including the present case, four adults (≥18 years) with 3q13.31 microdeletions have been reported in the literature to date (Table 1) [1,2]. Several features present in this patient, including DD/ID, speech delay, enuresis, postnatal growth above the mean, structural brain abnormalities, high arched palate, and skeletal anomalies, are consistent with previous reports of adults with 3q13.31 microdeletions (Table 1) [1,2]. Of all forty-one cases previously in the literature, the 3q13.31 deletion was reported as occurring de novo in thirty-eight (92.7%) and of unknown inheritance in three (7.3%) [1-4,14,15].

**Table 1:** Clinical features of four adults with 3q13.31 deletions

	**Current report**	**Molin et al. Case 1**	**Molin et al. Case 8**	**Shuvarikov et al. Case 6**
**Age (years)**	41	19.5	18	42
**Sex**	Male	Male	Male	Female
**Ethnicity**	European	NR	NR	NR
**OFC (percentile)**	97^th^	90^th^	97^th^	50-75^th^
**Height (percentile)**	75^th^-90^th^	95^th^	99^th^	10-25^th^
**Weight (category)**	Overweight	NR	NR	Obese
**Neurocognitive**	Borderline intellectual disability (VIQ 90, PIQ 62)	[Developmental delay]	[Severe intellectual disability] (FSIQ < 50)	[Borderline intellectual disability] (VIQ 94, PIQ 61)
**Speech delay**	Yes	No	Yes	Yes
**Neuropsychiatric**	[Schizophrenia], aggressive behaviour	ADHD	ADHD, some repetitive behaviours	Social and emotional immaturity
**Neurologic**	Delayed motor development, focal hypoplasia of the superior cerebellar vermis (15 y), enuresis	Hypotonia	Hypotonia	Cerebellar agenesis, EEG abnormalities, hypotonia, enuresis
**Musculoskeletal**	Bilateral L5-S1 disc protrusion, slight kyphosis	Kyphosis	NR	NR
**Genitalia**	NA	Small testes (8 ml)	Normal	Small introitus
**Craniofacial**	Broad neck, facial asymmetry, strabismus, narrow palpebral fissures, long philtrum, narrow high arched palate, flat occiput, frontal and occipital hair whorls (Figure 1)	Dolichocephaly, prominent broad forehead, strabismus, myopia, ptosis, antimongoloid slant, short philtrum, high arched palate, large ears, crowded teeth, soft enamel	Prominent/broad forehead, high arched palate, large fleshy ears, pointed chin	Absent eyebrows, epicanthal folds, down slanting palpebral fissures, ptosis, high palate, nystagmus, microstomia, small teeth, large palatine tori
**Hands and feet**	Cubitus valgus, bilateral short 4^th^ and 5^th^ metacarpals, narrow feet with mild bilateral clinodactyly and camptodactyly of toes	Small hands, long fingers	Tapering fingers	Pes cavus, middle finger clinodactyly bilaterally
**Pregnancy and birth**	Uneventful pregnancy, birth weight 3941 g (75^th^-90^th^ percentile)	NR	Birth weight 3430 g (50-85^th^ percentile)	Placental calcifications, marginally small for dates
**Other**	Insulin dependent Type 2 diabetes mellitus, hypercholesterolemia, hypocalcemia, hypertension	NR	NR	Large angioma in right shoulder, dorsocervical fat pad

OFC, occipital frontal circumference; NR, not reported; NA, not assessed; FSIQ, full scale intelligence quotient; VIQ, verbal intelligence quotient; PIQ, performance intelligence quotient; ADHD, attention deficit hyperactivity disorder; EEG, electroencephalography. Square brackets used to highlight ascertainment feature.

Neuropsychiatric phenotypes (i.e., ADHD and ASD) have been previously associated with the 3q13.31 microdeletion; however, this is the first report of schizophrenia in an individual with a 3q13.31 microdeletion. Moreover, the patient described in this report had a >25 point discrepancy between his performance and verbal IQ scores, consistent with a nonverbal learning disability [16]. Interestingly, the 42 year old female (Case 6) reported in Shuvarikov et al. [2] demonstrated a similar trend in IQ scores (Table 1). Raw IQ scores were not given for many of the paediatric cases, however Vuillaume et al. [4] described an affected 16 year old female (Case 1) as “being able to read and write but educational learning was difficult,” potentially describing a nonverbal learning disability. Detailed neuropsychological phenotyping of additional cases will help determine if nonverbal learning disabilities are part of the emerging 3q13.31 microdeletion syndrome.

### Candidate genes for neuropsychiatric expression

The four genes (*GAP43, DRD3*, *LSAMP,* and *ZBTB20*) that have been posited as contributing to the brain phenotype of the 3q13.31 microdeletion syndrome are all overlapped by this patient's deletion (Figure 2) [1,2]. Regarding these genes and their potential role in schizophrenia and related neuropsychiatric conditions, non-synonymous point mutations in *GAP43* were recently identified in two unrelated schizophrenia cases in a next-generation sequencing study [17]. *DRD3* has been a longstanding candidate gene for schizophrenia, largely based on its affinity to bind antipsychotic drugs and its localization in limbic brain structures [18]. Further, *LSAMP* and *ZBTB20* have each been implicated in various brain regions associated with schizophrenia. In a post-mortem study of schizophrenia, *LSAMP* expression was noted to be increased by ~20% in the dorsolateral prefrontal cortex of individuals with schizophrenia compared to controls [19]. In mice, *ZBTB20* knockdowns were noted to have faulty hippocampal cytoarchitecture and selective ablation of *ZBTB20* in mature hippocampal CA1 neurons led to disruptions in learning and memory [20,21].

In addition to genic haploinsufficiency, other molecular mechanisms may contribute to the phenotype of the 3q13.31 microdeletion syndrome. In particular, recent reports suggest miRNAs may play a role in mediating the risk for neurodevelopmental disorders [22,23]. Both miRNAs overlapped by the 3q13.31 deletion in this patient (miR-4796, miR-568) have predicted targets that are additional candidate genes for schizophrenia and related disorders, including *SHANK2* and *FMR1*[24,25]. The individual and collective influence of the above mentioned genes and miRNAs on the neuropsychiatric expression of the 3q13.31 microdeletion is deserving of further study. Of interest with respect to genotype-phenotype correlations are the few reports to date of the reciprocal 3q13.31 duplication, in which individuals appear to share some (i.e., DD/ID, hypotonia) but not all of the same clinical features as the 3q13.31 microdeletion [4,26]. The fact that neither deletions nor duplications at 3q13.31 were identified in 26,826 controls suggest that copy number aberrations in this region, rather than haploinsufficiency alone, may be associated with the deleterious phenotypic consequences.

### Implications for clinical practice

Cytogenetic anomalies may be found in up to 5-8% of cases with schizophrenia, suggesting a potential future role for clinical microarray testing in this population [9]. The occurrence of schizophrenia in the patient reported here could be unrelated to the 3q13.31 microdeletion. However, multiple lines of evidence, including the variable expression of many other recurrent large, rare CNVs, suggest a genetically-related neuropsychiatric spectrum of disease that includes both DD/ID and schizophrenia [9,27,28]. This is, to our knowledge, the first report of a 3q13.31 deletion discovered in a schizophrenia cohort. This attests to the overall rarity of these variants, and to the relative paucity of available data (almost all of which are research-based) compared with diseases like DD/ID where clinical microarray testing is now the first-tier diagnostic test [9,29]. More data are needed to delineate the role of pathogenic CNVs in the dual-diagnosis (schizophrenia and premorbid ID) population to which this patient would belong, where the yield may be significantly higher and where clinical testing is already indicated [30].

## Conclusions

In conclusion, we have identified an adult male with schizophrenia and a 3q13.31 deletion overlapping four promising neurodevelopmental candidate genes: *DRD3, ZBTB20, GAP43,* and *LSAMP*. Later-onset conditions like schizophrenia are increasingly associated with rare CNVs. Detailed phenotypic information across the lifespan facilitates genotype-phenotype correlations, accurate genetic counselling, and anticipatory care.

## Consent

Written informed consent was obtained from the patient for publication of this Case report and any accompanying images. A copy of the written consent is available for review through the Editor-in-Chief of this journal.

## Abbreviations

DD/ID: Developmental delay/intellectual disability; ASD: Autism spectrum disorder; ADHD: Attention deficit hyperactivity disorder; SRO: Shortest region of overlap; CNV: Copy number variation; CT: Computed tomography; IQ: Intelligence quotient; WASI: Wechsler Abbreviated Scale of Intelligence; CLIA: Clinical Laboratory Improvement Ammendments; miRNA: microRNA; TCAG: The Centre for Applied Genomics.

## Competing interests

SWS is on the Scientific Advisory Board of Population Diagnostics, Inc. and is a co-founder of YouNique Genomics. The other authors declare no conflicts of interest.

## Authors’ contributions

CL organized the clinical data and drafted and revised the manuscript. RM organized the clinical data for the manuscript. DJS carried out the clinical cytogenetic studies. ACL, CRM, and SWS carried out the molecular studies. GC carried out the study and helped draft and revise the manuscript. ASB conceived, designed, carried out the study, reviewed the detailed phenotypic data, and helped draft the manuscript. All authors read and approved the final manuscript.

## Supplementary Material

Additional file 1: Table S1 Genome-wide copy number variation detected by Affymetrix 6.0 microarray in the proband with a 3q13.31 deletion described in this report. Click here for file

Additional file 2: Table S2 Overview and negative results of 13 control datasets (total n=26,826) searched for 3q13.31 deletions and duplications. Click here for file
