# Supplementary material for: Adult expression of a 3q13.31 microdeletion
Source: Mol Cytogenet. 2014 Mar 20;7:23. doi: 10.1186/1755-8166-7-23 (PMC4022390; doi:10.1186/1755-8166-7-23)
Supplement: Additional file 1: Table S1 — Genome-wide copy number variation detected by Affymetrix 6.0 microarray in the proband with a 3q13.31 deletion described in this report. [file 1755-8166-7-23-S1.docx]

**Additional file 1: Table S1. Genome-wide copy number variation**^a^ **detected by Affymetrix 6.0 microarray in a proband with a 3q13.31 deletion.**

| **No.** | **Cytoband** | **Start**  **(build 36)** | **Stop**  **(build 36)** | **Estimated size (bp)** | **CN type** | **Rare** | **Class**^c^ |
| --- | --- | --- | --- | --- | --- | --- | --- |
| 1^b^ | **3q13.31** | **115,308,450** | **117,370,859** | **2,062,410** | **Loss** | **Yes** | **Pathogenic** |
| 2 | 2q14.3 | 123,879,663 | 123,925,928 | 46,266 | Gain | Yes | Uncertain clinical significance-likely benign |
| 3 | 14q31.3 | 84,302,948 | 84,356,082 | 53,135 | Gain | Yes | Uncertain clinical significance-likely benign |
| 4 | 1p36.21 | 12,768,450 | 12,906,093 | 137,644 | Loss | No | Benign |
| 5 | 1p31.1 | 72,541,504 | 72,583,736 | 42,233 | Loss | No | Benign |
| 6 | 1p13.3 | 111,179,088 | 111,189,749 | 10,662 | Loss | No | Benign |
| 7 | 1q21.1 | 147,303,148 | 147,526,040 | 222,893 | Loss | No | Benign |
| 8 | 1q44 | 246,815,817 | 246,863,836 | 48,020 | Loss | No | Benign |
| 9 | 2p16.3 | 52,607,984 | 52,635,046 | 27,063 | Gain | No | Benign |
| 10 | 3p21.1 | 53,003,415 | 53,016,559 | 13,145 | Loss | No | Benign |
| 11 | 3q26.1 | 163,995,351 | 164,109,297 | 113,947 | Loss | No | Benign |
| 12 | 2p12 | 81,379,746 | 81,412,688 | 32,943 | Loss | No | Benign |
| 13 | 3p14.2 | 61,453,717 | 61,465,189 | 11,473 | Loss | No | Benign |
| 14 | 4p15.2 | 25,578,592 | 25,591,051 | 12,460 | Loss | No | Benign |
| 15 | 4p16.1 | 9,070,328 | 9,088,443 | 18,116 | Gain | No | Benign |
| 16 | 4q13.1 | 59,656,666 | 59,670,704 | 14,039 | Gain | No | Benign |
| 17 | 4q13.2 | 69,057,536 | 69,168,574 | 111,039 | Gain | No | Benign |
| 18 | 5p15.31 | 7,230,338 | 7,242,925 | 12,588 | Loss | No | Benign |
| 19 | 5p15.2 | 12,868,780 | 12,879,650 | 10,871 | Loss | No | Benign |
| 20 | 5q15 | 97,074,222 | 97,125,076 | 50,855 | Loss | No | Benign |
| 21 | 5q33.2 | 155,409,350 | 155,427,837 | 18,488 | Loss | No | Benign |
| 22 | 5q35.3 | 180,311,316 | 180,350,709 | 39,394 | Loss | No | Benign |
| 23 | 6p25.3 | 202,353 | 326,149 | 123,797 | Loss | No | Benign |
| 24 | 6p21.33 | 31,394,255 | 31,404,430 | 10,176 | Loss | No | Benign |
| 25 | 6q14.1 | 77,496,587 | 77,509,523 | 12,937 | Loss | No | Benign |
| 26 | 6q16.3 | 103,844,669 | 103,868,754 | 24,086 | Loss | No | Benign |
| 27 | 7q34 | 141,416,112 | 141,438,576 | 22,465 | Loss | No | Benign |
| 28 | 7q34 | 142,156,294 | 142,167,486 | 11,193 | Gain | No | Benign |
| 29 | 8p23.1 | 6,976,336 | 7,877,252 | 900,917 | Gain | No | Benign |
| 30 | 8p11.23,8p11.22 | 39,354,760 | 39,506,122 | 151,363 | Loss | No | Benign |
| 31 | 9p21.3 | 23,353,115 | 23,363,484 | 10,370 | Loss | No | Benign |
| 32 | 9p21.3 | 24,487,680 | 24,507,682 | 20,003 | Loss | No | Benign |
| 33 | 11p15.1 | 18,905,648 | 18,918,564 | 12,917 | Gain | No | Benign |
| 34 | 11q11 | 54,722,184 | 54,793,048 | 70,865 | Loss | No | Benign |
| 35 | 12p13.31 | 9,525,137 | 9,619,559 | 94,423 | Gain | No | Benign |
| 36 | 13q21.33 | 68,146,300 | 68,166,243 | 19,944 | Loss | No | Benign |
| 37 | 14q11.2,14q11.1 | 18,842,018 | 19,493,212 | 651,195 | Gain | No | Benign |
| 38 | 14q21.2,14q21.3 | 42,896,392 | 43,304,724 | 408,333 | Gain | No | Benign |
| 39 | 14q32.33 | 105,612,798 | 105,638,145 | 25,348 | Gain | No | Benign |
| 40 | 17p11.2 | 18,296,117 | 18,405,946 | 109,830 | Gain | No | Benign |
| 41 | 17q12 | 31,464,091 | 31,509,204 | 45,114 | Gain | No | Benign |
| 42 | 17q21.31,17q21.32 | 41,756,832 | 42,107,479 | 350,648 | Gain | No | Benign |
| 43 | 19q13.12 | 40,541,333 | 40,553,688 | 12,356 | Loss | No | Benign |
| 44 | 19q13.32 | 50,513,025 | 50,595,314 | 82,290 | Gain | No | Benign |
| 45 | 19q13.41 | 58,621,576 | 58,706,990 | 85,415 | Gain | No | Benign |
| 46 | 22q11.23 | 22,680,529 | 22,726,814 | 46,286 | Gain | No | Benign |
| 47 | Xq21.32 | 92,209,788 | 92,224,260 | 14,473 | Gain | No | Benign |

Cytoband, cytogenetic location of copy number variation (CNV); CNV start and stop, hg18 (NCBI Build 36.1, March 2006); Estimated size, in base pairs; CN type, type of copy number aberration; Rare, yes if found in <0.1% of 2,357 population-based controls (see text and Costain et al., *Hum Mol Genet*, 2013 for details).

^a^CNV calls detected by at least two of three CNV calling algorithms (Birdsuite, iPattern, and Affymetrix Genotyping Console), and spanning at least 10 kb in length and five or more consecutive array probes (see Costain et al., *Hum Mol Genet*, 2013 for details).

^b^Previously reported by our group (Costain et al., *Hum Mol Genet*, 2013).

^c^Per the American College of Medical Genetics guidelines for CNV interpretation (Kearney et al., *Genet Med*, 2011).
