# Supplementary material for: Adult expression of a 3q13.31 microdeletion
Source: Mol Cytogenet. 2014 Mar 20;7:23. doi: 10.1186/1755-8166-7-23 (PMC4022390; doi:10.1186/1755-8166-7-23)
Supplement: Additional file 2: Table S2 — Overview and negative results of 13 control datasets (total n=26,826) searched for 3q13.31 deletions and duplications. [file 1755-8166-7-23-S2.docx]

**Additional file 2: Table S2. Control datasets examined for overlapping copy number variations at chromosome 3q13.31.**

| **Control dataset** | **# individuals** | **Array type** | **Description of dataset** | **Geographic region** | **# overlapping CNVs at 3q13.31**^a^ |
| --- | --- | --- | --- | --- | --- |
| POPGEN controls | 1,123 | Affymetrix 6.0 | Krawczak et al. 2006 (Community Genetics) | Germany | 0 |
| Ottawa Heart Institute controls | 1,234 | Affymetrix 6.0 | Stewart et al. 2009 (Journal of the American College of Cardiology) | Ontario, Canada | 0 |
| HapMap (Phase 3) controls | 1,056 | Affymetrix 6.0 | Altshuler et al. 2010 (Nature) | HapMap populations | 0 |
| Wellcome Trust Case Control Consortium (WTCCC) controls | 4,783 | Affymetrix 6.0 | Craddock et al. 2010 (Nature) | United Kingdom | 0 |
| Ontario Population Genomics Platform (OPGP) controls | 416 | Affymetrix 6.0 | Costain et al. 2013 (Human Molecular Genetics) | Ontario, Canada | 0 |
| Starr County Diabetes cases & controls | 1,794 | Affymetrix 6.0 | Below et al. 2011 (Diabetologia) | USA | 0 |
| Geneva NHS/HPFS Diabetes cases & controls | 5,966 | Affymetrix 6.0 | Qi et al. 2010 (Human Molecular Genetics) | USA | 0 |
| SAGE consortium controls | 1,287 | Illumina 1M | Bierut et al. 2010 (PNAS) | USA | 0 |
| Health, Aging, and Body Composition (Health ABC) Study controls | 2,566 | Illumina 1M-Duo | Coviello et al. 2012 (PLoS Genetics) | USA | 0 |
| Ontario ARCTIC controls | 1,120 | Affymetrix 500k | Zogopoulos et al. 2007 (Human Genetics) | Ontario, Canada | 0 |
| Itsara et al. controls | 2,493 | Illumina Hap550, Hap650Y and Hap300 | Itsara et al. 2010 (American Journal of Human Genetics) | Populations across the world | 0 |
| KORA | 1,775 | Illumina Omni 2.5M | Verhoeven et al. 2013 (Nature Genetics) | Germany | 0 |
| COGEND | 1,213 | Illumina Omni 2.5M | Bierut et al. 2007 (Human Molecular Genetics) | USA | 0 |
| **Total** | **26,826** |  |  |  | **0** |

^a^Number of CNVs (losses and gains) overlapping the rare 3q13.31 microdeletion described in this report (chr3:115,308,450-117,370,859; hg18), using a 50% reciprocal overlap criterion (see Lionel et al., *Sci Transl Med*, 2011 for details)
